# Overview of C3 Glomerulopathy

**DOI:** 10.3389/fped.2016.00045

**Published:** 2016-05-06

**Authors:** Vimal Master Sankar Raj, Roberto Gordillo, Deepa H. Chand

**Affiliations:** ^1^University of Illinois College of Medicine, Peoria, IL, USA; ^2^Associate Medical Director, Research and Development, Abbvie, Chicago, IL, USA

**Keywords:** C3 glomerulopathies, atypical HUS, complement C3, kidney disease, children

## Abstract

C3 glomerulopathy is an umbrella term, which includes several rare forms of glomerulonephritis (GN) with underlying defects in the alternate complement cascade. A common histological feature noted in all these GN is dominant C3 deposition in the glomerulus. In this review, we will provide an overview of the complement system as well as mediators, with an introduction to pharmaceutical agents that can alter the pathway.

## Background

Membranoproliferative glomerulonephritis (MPGN) is a histopathological pattern of renal injury characterized by thickening of capillary walls and mesangial enlargement secondary to increased cellularity and matrix deposition. Traditional classification of MPGN is based on morphological features seen in electron microscopy (EM) and classified into type I (mesangial and subendothelial electron dense deposits), type II (electron dense material in the glomerular basement membrane), and type III (subepithelial deposits with basement membrane spikes). But an improved understanding of the pathogenic mechanism in MPGN has led to an emergence of a new grouping of diseases known as the C3 glomerulopathy (C3G). Based on IF findings, a more pathogenic reclassification of MPGN has been proposed into the following three subtypes (1): (i) Ig-associated MPGN, (ii) MPGN with dominant C3, and (iii) idiopathic MPGN (not C3G or Ig associated). This new classification system enables to outline diagnostic evaluation in various subgroups. The subgroup “MPGN with dominant C3” is helpful in identifying patients who would require an investigation of the complement pathway. The utility of this reclassification system was confirmed in a pediatric study (2) where C3G was shown to be more resistant to immunotherapy when compared to classical MPGN.

Examination of the mechanisms involved in uncontrolled C3 activation in the affected families has given important insight in the understanding of the pathophysiology of disease. Many of the factors involved in complement dysregulation in C3GN are very similar to that of atypical hemolytic uremic syndrome (aHUS). Though the pathophysiology is very similar, aHUS has a more systemic presentation and can practically involve all the organ systems while the damage in C3GN is localized to the kidneys. In this review, we will provide an overview of the complement system as well as mediators, with an introduction to pharmaceutical agents that can alter the pathway.

## Overview of the Complement System

**Figure 1:**
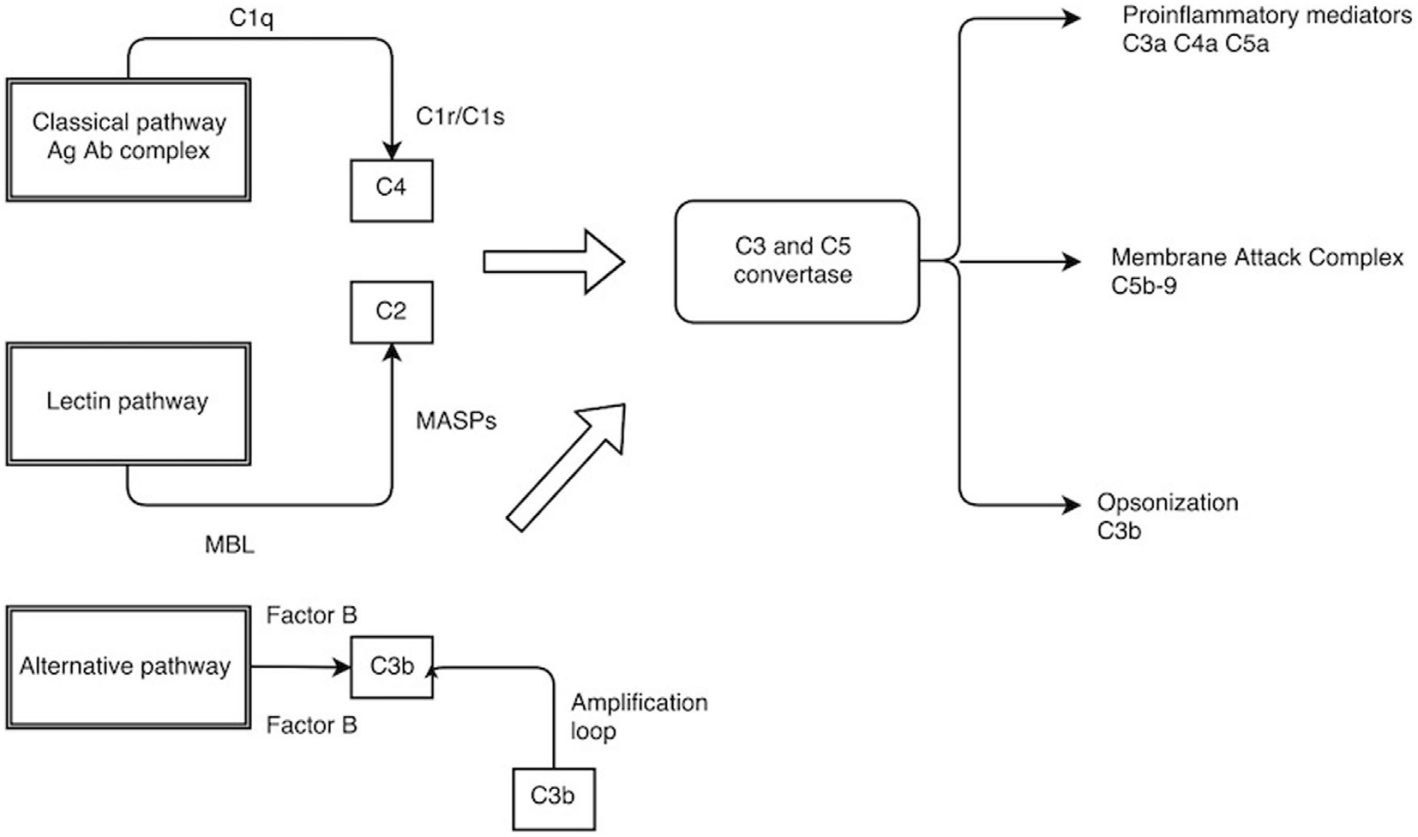
**The complement pathway**. C1q, C1r/C1s–C1 complex; MBL, mannose-binding lectin; MASPs, MBL-associated serine protease.

The complement system is a central part of the innate immunity and involves more than 30 proteins. The complement cascades have specific roles starting from identification of foreign organism, generation of potent inflammatory mediators (anaphylatoxins), coating of the pathogenic surface (opsonization), and targeted lysis by the formation of membrane penetrating pores, namely, the membrane attack complex (MAC) (3). There are three arms to the complement system (Figure 1):

A: classical pathway – activated by antigen–antibody immune complex,
B: lectin pathway – activated by the binding of mannose-binding lectin (MBL) to the mannose residues on the bacterial surface,
C: alternative pathway – constitutively active under the inhibitory activity of complement regulators.

It is important to note that all three pathways converge at the point of C3 cleavage and have a common effector phase. This involves accumulation of proinflammatory molecules (C3a, C4a, and C5a) of complement cleavage, potent opsonin in C3b, and ultimate lysis of the target cell using MAC (C5b–9).

The classical pathway becomes activated when the Fc component of the antigen–antibody immune complex binds to the C1 complex, which is a combination of C1q and serine proteases (C1r and C1s). Autocatalytic activation of serine protease ensues and cleaves C4 and C2 to form the classic C3 convertase (C4bC2a) (4, 5).

The lectin pathway employs pattern recognition receptors (PRR) such as MBL to recognize microorganisms by means of highly conserved structures known as pathogen-associated molecular pattern (PAMPs) (6). Some examples of PAMP include lipoteichoic acid in Gram-positive bacteria; lipopolysaccharide in Gram-negative bacteria; and β glucan in fungi. Once the MBL recognize these PAMPs, it activates the serine protease associated with it (MASP), which then cleaves C4 and C2 in a manner very similar to the classic pathway.

The alternative pathway, which is continually active, generates C3b, which can bind indiscriminately to both host cells and foreign microbial surfaces. On a foreign surface such as bacterium, C3b binds with its activator complement factor B (CFB) that is then cleaved by factor D (CFD) to form the alternative C3 convertase (C3bBb). The C3 convertase thus formed enters into an amplification loop exponentially cleaving further C3. The final end product is the formation of C5 convertase (C3bBbC3b), which participates in the assembly of MAC C5b–9 (MAC). C5b forms the basis for the MAC assembly and associates with C6, C7, C8, and C9 on the target cell surface. This association enables C9 to form a stable pore up to 10 nm in diameter on the cell membrane. A rapid rise in intracellular calcium along with loss of mitochondrial polarity and adenine nucleotide pools will result in cellular death either by necrosis or apoptosis (7–9).

**Figure 2:**
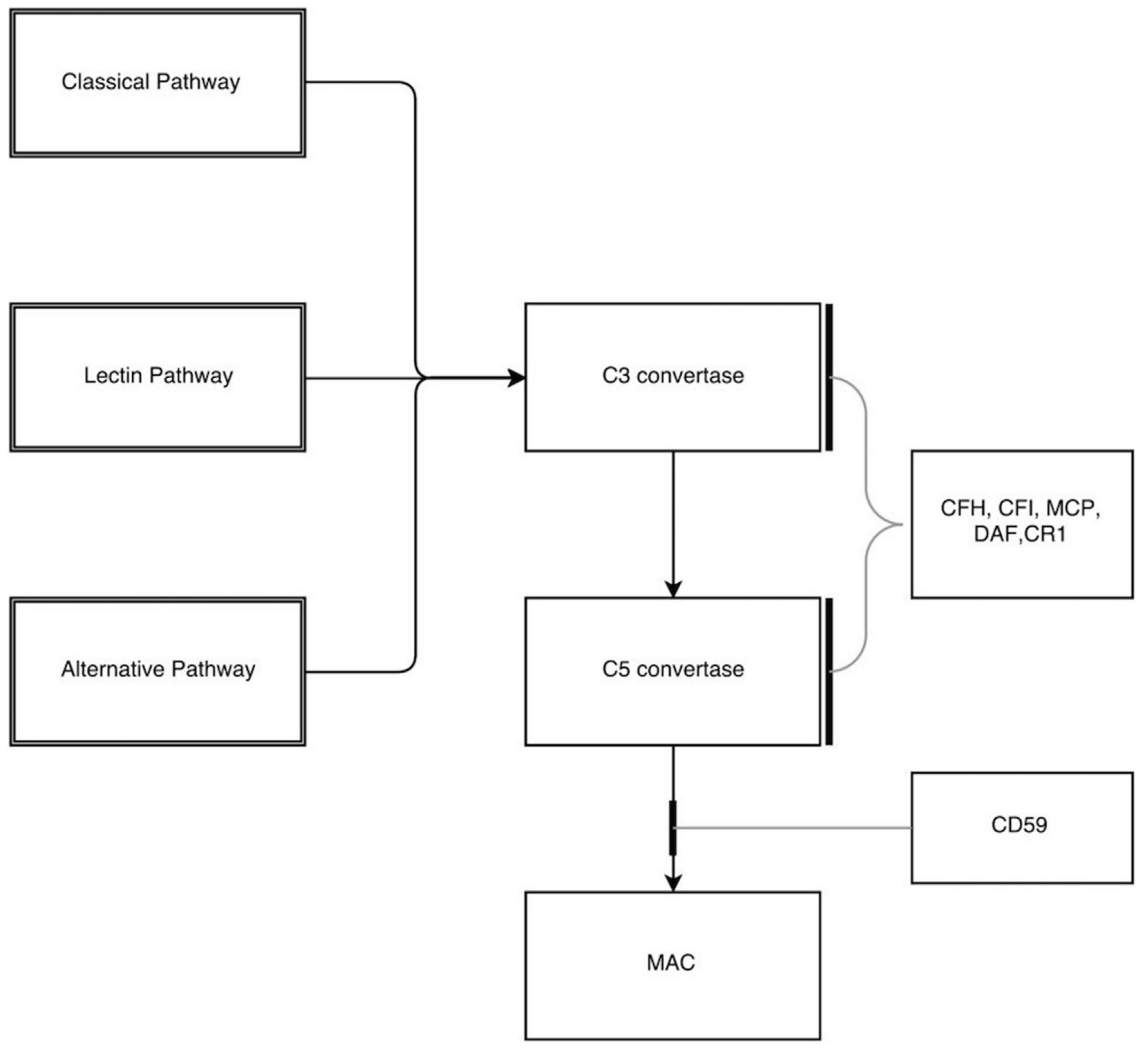
**Inhibitors in the complement pathway**. CFH, complement factor H; CFI, complement factor I; MCP, membrane cofactor protein; DAF, decay-accelerating factor; CR1, complement receptor 1; MAC, membrane attack complex.

There are regulatory mechanisms in place that prevent the uncontrolled activation of the alternative pathway and hence protecting host cells from damage to self. The complement regulators act mainly by the following mechanisms:

- Prevent the formation of active convertase.
- Complement decay-accelerating factor, also known as CD55 or DAF is a 70-kDa membrane protein that regulates the complement system on the cell surface. DAF recognizes C4b and C3b fragments that are created during classical and alternate pathway activation and play a main role in accelerating the decay of active convertase once formed.
- Complement Factor H, a large soluble glycoprotein, plays a critical role in the homeostasis of complement system. It also contributes to essential cofactor activity for the regulatory function of both Factor I and DAF.
- Factor I is a serine protease, which plays a main role in the catabolism of C4b and C3b into inactive fragments thereby preventing the formation of active convertase. Factor I requires a number of cofactors for this regulatory action, namely, membrane cofactor protein (MCP) along with Factor H, complement 4 binding protein (C4BP), and complement receptor 1 (CR1) (10). DAF in addition to other coregulatory proteins (Factor H, CR1, and C4BP) primarily inhibits the formation of new convertase and shortens the half-life of preformed convertase (11). The final level of control is to inhibit the assembly of MAC itself through inhibitors such as CD59, vitronectin, and S protein. Proteins regulating the complement cascade are present in both fluid (plasma) and membrane phase (cell surface) ensuring that complement activation is mainly targeted to remove the invading pathogen and avoiding uncontrolled activation (Figure 2).

## Key Components of the Complement Cascade

### Complement Factor H

Complement Factor H (CFH) is the most important protein involved in the regulation of alternate pathway. CFH is a single polypeptide chain of glycoprotein synthesized primarily in the liver and consists of 20 short consensus repeats (SCR) with two C3b-binding sites (12). SCR 1–4 in the amino terminal mainly acts on the fluid phase binding to C3b. SCR 7, 19, and 20 have binding sites, which attach to the glycosaminoglycan (GAG) on the cellular endothelial surface, thus helping to regulate the alternate pathway in the membrane phase. SCR 19 and 20 in the carboxy terminal acts at the membrane phase by anchoring CFH to C3b bound to GAG/sialic acid on the host cell surface while SCR7 acts as a secondary anchor (13). In addition, CFH is the required cofactor for CFI-mediated cleavage of C3b to inactivated C3b.

Mutations in CFH were the first identified abnormality in alternate complement cascade. Familial studies were helpful in mapping CFH and MCP to the RCA locus in 1q32. The initial reported CFH mutation was a heterozygous defect in SCR20 detected in a patient with atypical HUS (14). Subsequently, several mutations of the CFH have been detected mostly in the SCR 19 and 20. More than 50% of the mutations have been mapped to SCR20.

Though some mutations can present with a quantitative deficiency with decreased plasma CFH levels (Type 1 mutations), majority of mutations especially involving SCR 19 and 20 are functional defects with normal plasma CFH levels (type 2 mutations). Structurally related proteins to CFH, namely, CFH-related proteins (CFHR1–5) are identified in the human plasma, the functional significance of which is not known. As the genes for CFH and CFHR are in close proximity, genetic rearrangement between homologous sequences can result in a hybrid CFH–CFHR protein, which has lost the SCR 19 and 20. The hybrid protein has altered function and can cause complement pathway dysregulation with or without causing quantitative reduction in plasma levels of CFH and C3. C3 is noted to be decreased in about 30–50% of heterozygous mutations.

Acquired dysfunction of CFH due to antibodies will also result in alternate pathway dysregulation. In about 90% of patients with anti CFH antibodies, complete deficiency of CFHR1 and CFHR3 is noted secondary to large deletions of these genes (15).

### Membrane Cofactor Protein

Membrane cofactor protein is a transmembrane glycoprotein expressed in all host cells except for red blood cells. MCP contains an extracellular amino terminal, which has four SCR followed by the transmembrane portion and the cytoplasmic anchor. MCP acts as the main cofactor at the membrane level for CFI action of cleaving C3b with the C3b binding sites located to SCR 2, 3, and 4. Like CFH, MCP is located in the RCA gene cluster at 1q32. Atypical HUS patients with MCP mutation have decreased expression of MCP on peripheral leukocytes though functional defects have been noted rarely (16). Of note, as MCP is a membrane phase regulator of the alternate pathway, C3 level measurements will usually be normal. A concomitant decrease in C3 levels with MCP mutation may point toward a coexisting mutation in a fluid phase regulator.

### Gain of Function Mutations

In contrast to mutations in the inhibitory factors, in which negative control of the alternate pathway is abnormal, a gain of function mutation results in persistent activation of the alternate pathway. They usually present with low C3 levels and normal C4 levels indicating alternate pathway activation.

### Complement Factor B

Complement factor B (CFB) is a zymogen and contains catalytic site for the formation of alternate C3 convertase. CFB on binding with C3b is cleaved by complement factor D (CFD) into Ba and Bb. The cleaved portion Bb combines with C3b to form C3bBb, which is the alternate C3 convertase which in turn, cleaves C3 further, resulting in an amplification loop. A gain of function mutation in CFB induces increased activity and stability of C3 convertase (17). With persistent activation of alternate pathway, C3 levels will be low in these patients.

### C3 Mutations

Heterozygous C3 mutations were first described in 2008 in aHUS patients with persistent low C3 levels (18). *In vitro* assessment of these mutated C3 showed a decreased ability to interact with MCP resulting in an indirect gain of function mutation. Plasma C3 levels are usually decreased in these patients.

### Dense Deposit Disease

Dense deposit disease (DDD), once classified as MPGN type 2, derives its name from the electron dense deposits noted by EM in the lamina densa of the glomerular basement membrane (GBM) (19). Light microscopy (LM) presentation is varied and can present with varying degrees of mild mesangial proliferation, endocapillary proliferation to the classic MPGN like picture with essential defining feature being the dense deposit transformation of the GBM (20, 21). Immunofluorescence (IF) shows predominant C3 deposition along with its breakdown products in the glomerular basement membrane.

Insight into the mode of alternate complement pathway dysregulation in DDD comes from familial studies. Martínez-Barricarte et al. (22) noted in a family of a mother and her two identical twin boys, a mutation characterized by a two amino acid deletion in C3. The mutant C3 in turn led to the formation of a hydrolyzed mutant C3 convertase. The mutant C3 convertase was resistant to degradation by fluid phase regulators (CFH) and was capable of cleaving wild type C3 with sustained fluid phase activity. Importantly, the mutation in the C3 convertase did not affect its ability to be regulated by surface regulators, such as decay-accelerating factor (DAF). This implies that the fluid phase dysregulation is the main abnormality noted in this particular familial DDD and sMAC is not usually elevated.

C3 nephritic factor (C3NeF) is an autoantibody that binds to the C3 convertase in the fluid phase stabilizing it against the cleaving action of CFH. The net result is uncontrolled activation of C3 with low C3 levels. C3NeF though commonly seen in DDD is not specific and has also been noted in MPGN type 1 and C3GN (23, 24). Other noted pathology includes autoantibodies against CFH in some patients with DDD (25, 26). The common denominator noted in different mechanisms leading to DDD is fluid phase dysregulation.

Dense deposit disease is usually diagnosed in children but has been noted in adults as well. The usual presenting features are proteinuria, hematuria, hypertension, and renal failure. Low serum C3 is a common finding. Progression to ESRD is noted in almost 50% of patients (27). Ocular drusen (28), which is an ocular lipoproteinaceous complement debris deposit and acquired partial lipodystrophy (APL), can be associated with DDD.

A significant complication of DDD is disease recurrence in transplant recipients leading to allograft failure (29).

### C3 Glomerulonephritis

In C3 glomerulonephritis (C3GN), dominant deposition of C3 is noted in the mesangium and capillary wall. Both subepithelial and subendothelial deposits may be present, and by light microscopy can resemble MPGN or post-infectious GN. In contrast to DDD, intramembranous deposits, if found, are discontinuous without the osmiophilic, ribbon like appearance. The consensus report (2) classifies GN with dominant C3 deposition into (i) C3 glomerulopathy, (ii) Post-infectious GN, and (iii) other etiology. C3 glomerulopathy is further classified into C3GN and DDD based on specific genetic forms, autoantibodies, and electron micrographic findings.

The pathophysiology for alternate pathway activation in C3GN is very similar to DDD with fluid phase dysregulation due to varying mutations or autoantibodies. Familial studies have shed some important information such as the deletion of CFH codon noted in sisters from a consanguineous Turkish family with C3GN (30, 31). The result was a mutant CFH with defective binding to C3b (32). Various heterozygous mutation of CFH, CFI, and MCP genes have been reported in the French literature (33). Clinical presentation varies with different degrees of proteinuria, hematuria, and renal insufficiency. Renal survival was worse if the GFR at diagnosis is <60 ml/min/1.73 m^2^.

### Complement Factor H-Related Protein 5 Nephropathy

Complement factor H-related protein 5 (CFHR5) nephropathy is a subtype of C3GN with autosomal dominant inheritance, reported by Gale et al. (34) from two families in Cyprus. Though light microscopy and IF features were very similar to C3GN, genome wide linkage analysis localized the abnormality to 1q 31–32, a region that includes both CFH and CFHR genes.

Genes encoding CFH and CFHR proteins are in close proximity to each other within the RCA gene cluster of 1q32 with a high degree of sequence homology predisposing to duplication, deletion, and hybrid gene formation. CFHR5 is a 65-kDa plasma protein with nine SCR. Though its physiologic role is not completely understood, it has been elucidated that this protein does play a role in inhibiting C3 convertase activity (35, 36). In patients with CFHR5 nephropathy, duplication of exons 2 and 3 of the CFHR5 gene leads to a novel CFHR5 protein with 11 SCR. The mutant protein is less effective in binding with C3b and hence leads to dysregulation of the fluid phase of alternate pathway.

Clinical features can be subtle with proteinuria and microhematuria. Gross hematuria with upper respiratory infections (synpharngitic) similar to IgA nephropathy could be a presenting feature. Varying grades of renal dysfunction are usually present as well.

### Diagnosing C3 Glomerulopathy

A multi-pronged approach involving both structural and functional components is necessary to establish a diagnosis of C3 glomerulopathy. Structural diagnosis involves obtaining tissue *via* renal biopsy to demonstrate predominant C3 deposition. Assessing the functional component of alternate pathway dysregulation involves an extensive evaluation that includes measurement of complement levels, specifically C3, C4, complement factors (CFH, CFI, and CFB), C3NeF, and autoantibodies to CFH and CFB. In addition, genetic testing (direct exon sequencing) for mutations in varying complement regulators (CFH, CFI, MCP, CFB, and C3) may be needed. Assessment of copy number variations (CNV) across CFH–CFHR locus will be useful to detect hybrid genes.

### Renal Histology

Light microscopic findings in C3 glomerulopathy can range from membranoproliferative lesions to mesangioproliferative or endocapillary proliferative lesions with or without absence of crescents. In rare instances, light microscopy might be normal. The electron dense osmophilic deposits as seen characteristically in DDD are found within the glomerular basement membrane, and as rounded deposits in the mesangium. In many cases, deposits are also seen in Bowman’s capsule and tubular basement membranes. C3 glomerulopathy, in which deposits do not completely fulfill criteria for dense deposits, are classified as C3GN. EM in C3GN shows a complex pattern of mesangial increase and glomerular basement membrane thickening. Differing combinations of subendothelial, intramembranous, and subepithelial deposits are noted.

Immunofluorescence shows characteristic C3 fragment deposition in C3GN. Though looking for terminal MAC, C5b–9 may be relevant considering the anti C5 therapy with eculizumab, the terminal MAC is also found in normal glomeruli and tubular basement membrane making it an unreliable diagnostic marker. With inconsistencies in the reagents for detecting terminal complement components and with C5b–9 also noted in repeat biopsy of C3GN, years after treatment, the current diagnostic appeal of the terminal MAC is very limited (37). Current knowledge on the significance of differing pathologic findings on the clinical course and response to anti complement therapy is incomplete and is a topic for further research consideration.

### Treatment

Non-specific treatment measures include supportive therapy aimed at reduction of proteinuria and aggressive control of hypertension. Angiotensin-converting enzyme (ACE) inhibitors and angiotensin II receptor blockers are the first line agents prescribed to reduce proteinuria and improve renal hemodynamics (38). In the presence of hyperlipidemia, lipid lowering agents can also help delay progression of renal disease.

Plasma infusions may be beneficial in patients with known factor deficiency (31, 39). In patients with circulating antibodies (Anti CFH antibody or C3Nef), plasma exchange with fresh frozen plasma can be beneficial to remove the offending antibodies and replenish necessary complement factors (40–43). Plasma exchange or infusion should be performed at intervals keeping the half-life of the replenished factor in mind (44, 45). Immunosuppressive medications used in other forms of GN such as mycophenolate mofetil and rituximab have been tried with varying results (46, 47).

Disease-specific treatment with medications affecting the complement cascade such as eculizumab has been a topic of much discussion. The aim of such treatment is to prevent C5 cleavage. As such, eculizumab could provide a targeted therapy for patients with C3 glomerulopathy similar to its use in aHUS and paroxysmal nocturnal hemoglobinuria (PNH). Bomback et al. (48) conducted an open label non-blinded study using eculizumab in six adult subjects with C3 glomerulopathy for a total medication period of 53 weeks using aHUS protocol. Though all the subjects tolerated the medication well with no significant adverse effects, improvement in renal function was noted only in two of six subjects both of whom had elevated sMAC levels. Additional anticomplement therapies that could provide complement regulation at C3 convertase instead of C5 might be a useful targeted therapy.

The efficacy of targeted complement inhibition in experimental mouse model of C3G as shown by Ruseva et al. (49) has provided us with a glimpse of future therapeutic options. Experimental mouse models with C3G (CFH deficient or CFH/CFI deficient) were treated with a recombinant mouse protein (CR2–FH). CR2–FH is a fusion protein composed of complement regulatory domains of Factor H linked to C3 fragment-binding domain of complement receptor 2 (CR2). This unique fusion protein was able to bind C3 fragments at sites of complement activation and prevent further C3 activation. CR2–FH partially restored plasma C3 levels in CFH deficient mouse and also reduced the linear C3 reactivity along GBM. It also stopped *de novo* C3 deposition in GBM.

Currently, there is no serum or histological marker that could predict the nature of disease progression or response to therapy. The primary aim for therapy should be prevention of disease progression as monitored by periodic assessment of renal function and degree of proteinuria. With C3GN being a disorder of alternate pathway, the use of anticomplement therapy is logical but the role of alternate immunosuppression has not been clearly established. Supportive therapy with ACE inhibitors or angiotensin 2 receptor blockers continue to be used based on extrapolation from other proteinuric renal diseases. Questions over when to initiate anticomplement therapy and the duration of therapy have yet to be answered. An effort toward creating an international pathology registry as advised by the consensus report on C3 glomerulopathy (2) could provide important information regarding this rare disease. As understanding of the disease process improves and novel treatments (such as recombinant factor H) become available, more targeted disease therapy could be incorporated in treating this rare condition.

## Author Contributions

All authors listed, have made substantial, direct and intellectual contribution to the work, and approved it for publication.

## Conflict of Interest Statement

Dr. DC is an employee and shareholder at Abbvie. The remaining authors declare that the research was conducted in the absence of any commercial or financial relationships that could be construed as a potential conflict of interest.
